# Preparation and properties of novel binary and ternary highly amorphous poly(vinyl alcohol)-based composites with hybrid nanofillers

**DOI:** 10.1038/s41598-023-46083-2

**Published:** 2023-11-05

**Authors:** Anastasiia Stepura, Matej Mičušik, Federico Olivieri, Gennaro Gentile, Marino Lavorgna, Maurizio Avella, Edita Matysová, Jarmila Vilčáková, Mária Omastová

**Affiliations:** 1 Polymer Institute of Slovak Academy of Sciences, Dúbravská cesta 9, 845 41 Bratislava, Slovakia; 2 Institute of Polymers Composites and Biomaterials, National Research Council of Italy, Via Campi Flegrei 34, 80078 Pozzuoli (Naples), Italy; 3 Institute of Polymers Composites and Biomaterials, National Research Council of Italy, Piazzale Enrico Fermi 1, 80055 Portici (Naples), Italy; 4 SYNPO akciová společnost, S. K. Neumanna 1316, 532 07 Pardubice V, Czech Republic; 5 Faculty of Technology, Tomas Bata University in Zlín, Vavrečkova 5669, 760 01 Zlín, Czech Republic

**Keywords:** Materials chemistry, Polymer chemistry, Nanocomposites

## Abstract

Smart protective coatings and devices are currently of great interest. In particular, they can absorb or reflect harmful waves of electromagnetic interference (EMI). In this work, novel binary and ternary composites with highly amorphous poly(vinyl alcohol) (HAVOH) as a matrix and single-walled carbon nanotubes (SWCNTs) and MXenes as nanofillers were prepared. HAVOH is a recently patented kind of poly(vinyl alcohol) (PVOH) that was modified with diol monomers. MXenes are a new type of inorganic two-dimensional (2D) nanoparticle consisting of carbides, nitrides and carbonitrides. Three series of composites, HAVOH/SWCNTs, HAVOH/MXenes and HAVOH/SWCNTs/MXenes, were prepared using the solvent casting method. Samples were tested with various methods to study their structure, electrical properties, thermal behavior and EMI-shielding properties. HAVOH/3.0 wt.% SWCNTs/3.0 wt.% MXene specimens revealed a shielding effectiveness of 55 dB, which is 122 times better than that of the neat matrix. These results are promising for the fabrication of films with protective effects against EMI.

## Introduction

Demand for innovative polymeric composites is continually growing. The preparation of this class of materials, especially low-cost and high-performance polymeric composites, is still a challenge for researchers. An important factor is also the selection of the polymeric matrix. Most polymers require the utilization of some organic solvents for their dissolution, and large-scale production is often not environmentally acceptable. Thus, polymeric composites prepared with solvent casting using water-soluble materials have better opportunities for application. There are few water-soluble polymers produced on a large scale. Among them, one of the most relevant is poly(vinyl alcohol) (PVOH). Among different PVOH types, recently, a modified PVOH containing diol monomers named highly amorphous poly(vinyl alcohol) (HAVOH), trademark G-Polymer, was produced^1^. The main advantages of HAVOH over typical PVOH are its semicrystalline nature, excellent water solubility, good melt processing by extrusion and low oxygen permeability but poor water barrier resistance, which makes it hard to use in food packaging, requiring the utilization of additives as well as crosslinkers^2–4^. On the other hand, HAVOH can be used for polymeric nanocomposite preparation. As shown by Donato et al.^3^, HAVOH represents an effective matrix for the realization of multifunctional nanocomposites with good electrical, mechanical and thermal properties. Besides that, HAVOH was applied as a matrix for the preparation of composites with different fillers, such as multiwalled carbon nanotubes (MWCNTs)^5^, graphite^6^, graphene oxide^7^, clay^2^, silica^3^, cellulose^8^, and single-walled carbon nanotubes (SWCNTs)^9,10^. In some cases, it is difficult to find a solvent that is suitable for both HAVOH dissolution and filler dispersion, and specific methods are required to process the nanocomposite formulation. Santillo et al.^5^, who used MWCNTs as fillers, previously dispersed nanotubes in THF (tetrahydrofuran) with the addition of an ionic liquid, enabling them to interact with the selected filler. When HAVOH was added, a stable dispersion of polymer coated with filler was formed, as HAVOH was not soluble in the aforementioned solvent, but it formed hydrogen bonds with the applied ionic liquid (BenzImCl—1-Benzyl-3-methyl-imidazolium chloride). The prepared samples showed good electrical conductivity, 0.79 S/cm and were processed by 3D printing, obtaining effective EMI-shielding systems.

In the last decade rising number of different kinds of devices, expanding of areas (territories) of cover with wireless local area networks and radars, significantly increased the level of so-called "electric field pollution". It creates electromagnetic radiation due to radio waves and microwave radiation, which is emitted by all electronic devices, particularly those that operate in the radio wave and microwave range of frequencies (e.g., cell phones). Consequently, such phenomenon causes electrosmog. Of great concern is that the radiation interferes with electronics, due to the interaction of the electrons in the metal conductors with the electric field in the radiation^11^. Hence, it causes malfunctions of aforementioned devices and it can also cause human health problems. Therefore, currently researchers are highly engaged in creating a new class of materials called electromagnetic interference (EMI) shielding materials^12^. For good shielding effectiveness (SE) materials have to possess high electrical conductivity (EC). Pure metals such as copper, aluminum, stainless steel have an outstanding conductivity, thus prominent SE. However, they are expensive, with low flexibility, high density, and tend to easily corrode. Recently, nanoparticles become of great interest in the view of application in EMI-shielding area. This is due to higher aspect ratio, higher interfacial reactivity, and unique chemical and physical properties due to nanoscale sizes. Among others, 2D MXenes are recently discovered very promising family of inorganic nanoparticles. MXenes were first obtained and described in 2011 at Drexel University, USA^13,14^. The first article devoted to MXene discovery reported the preparation of Ti_3_C_2_T_x_ from the Ti_3_AlC_2_ MAX phase precursor^13^. MXenes consist of quite thin (only a few atoms) layers of transitional metal carbides, nitrides, and/or carbonitrides. The general formula of MXenes is M_*n*+*1*_X_*n*_T_*x*_, where M is an early transitional metal (Sc, Y, Ti, Zr, Hf, V, Nb, Ta, Cr, Mo, or W), X is carbon and/or nitrogen, and n = 1–3. T represents the surface termination groups that are mostly = O, -F, -OH, and x in T_x_ represents the number of surface functionalities. MXenes have an impressive list of properties, such as a high Young’s modulus^15^, good thermal and electrical conductivities^16,17^, adjustable band gap^18^, UV-light absorbance^19^, good EMI-shielding properties^20^, and large energy capacitance^21^. Due to possessing a variety of properties, MXenes are promising candidates for numerous applications, and among others, these nanoparticles could be used as fillers in polymeric nanocomposites^22^. Currently, more than 40 different MXenes have been prepared and described, and over 100 stable compounds have been theoretically predicted^23,24^.

MXenes possess few properties, which are favorable for their application as EMI-shielding barriers. First of all, their layered structure, as due to this, incident waves when entering the structure "interact with the high electron density of MXene, leading to an ohmic loss of EM waves"^25^, and later they are changed into multiple intrinsically reflected (absorbed) waves, thus significantly decreasing the number of transmitted waves. Secondly, MXenes high EC also plays an important role. It is advantageous in this application field as this means high number of free electrons and high-density electronic cloud. The presence of free electrons provides reflection of the incident electromagnetic waves. Additional properties, which make MXenes promising EMI-shielding material, are large specific surface area, which can be adjusted through synthesis conditions, lower density, comparing to heavy metals, ultralow thickness. Ti_3_C_2_T_x_ MXene was first investigated for its absorption performance in 2016, comparing it to that of the corresponding MAX phase (Ti_3_AlC_2_). The results showed that when a material thickness of 1.4 mm was used at a filling ratio of 50 wt.%, the extreme reflection loss of Ti_3_C_2_T_x_ was − 17 dB, which was much lower than that for the MAX phase^26^. The EMI shielding parameters of MXenes can be enhanced when combined with polymers, various types of fibers, carbon derivatives, metals, metal–organic frameworks and other materials, as described in published papers^27–29^. In the work of Shazad et al.^30^ MXenes were firstly studied in a composite for their EMI SE (shielding effectiveness) properties. They prepared Ti_3_C_2_T_x_—sodium alginate samples and received 57 dB of SE. Another excellent result was obtained by Liu et al.^31^, who fabricated flexible, hydrophobic MXene foams, which revealed outstanding 70 dB of EMI-shielding performance. Even better values were achieved by Nguyen et al.^32^. In this work authors prepared hybrid composites with introducing both MXenes nanoparticles and graphene foam (GF) into PDMS (poly(dimethylsiloxane)) polymer matrix. The highest average EMI SE was achieved with Fe_3_O_4_@Ti_3_C_2_T_x_/GF/PDMS sample reaching 80 dB in X-band, 77 dB in Ka-band, 83.6 dB at 8.7 GHz and 78.9 dB at 39.6 GHz. This specimen containing 11.5% of Fe_3_O_4_@Ti_3_C_2_T_x_ with thickness 1 mm showed also superb 630 S/cm of conductivity. Slightly lower results were obtained in the work of Song et al.^33^. Here were prepared also hybrid composite, however instead of graphene was used honeycomb structural reduced graphene oxide (rGH) and as a matrix was used epoxy resin. Addition of only 1.2 wt.% of rGH and 3.3 wt.% of MXenes led to receiving ~ 390 S/cm of electrical conductivity and 55 dB of shielding effectiveness. In the next work fabricated composites are close to those mentioned in this work in the viewpoint of polymer matrix. Jin et al.^34^ prepared flame-retardant multilayered films with MXene filler and PVA (poly(vinyl alcohol)). The 27-μm thick PVA/MXene film exhibited remarkable 716 S/cm of EC and ~ 44 dB of EMI SE. However, as much as 19.5 wt.% of filler load was used in this specimen. At the same time, composite revealed 23-fold enhanced thermal conductivity, comparing to neat PVA, what is a promising result for further application as films for prevention of flame propagation.

In this work, a new type of HAVOH-based polymeric nanocomposite was prepared by solution casting, mixing an aqueous solution of HAVOH with various amounts of delaminated 2D MXene in a water suspension. Moreover, nanocomposites containing a 1D filler, SWCNTs, were prepared by the same method. The combination of 1D and 2D fillers is an interesting approach for innovative polymeric composite creation with high application potential; therefore, a third series of HAVOH composites was prepared containing a hybrid mixture of MXenes and SWCNTs. Prepared nanocomposites were characterized using a multitechnique approach. Scanning electron microscopy (SEM) and transmission electron microscopy (TEM) were used to characterize the morphology and structure of the obtained nanocomposites, broadband dielectric spectroscopy (BDS) was used for electrical conductivity measurements. The application potential was examined by EMI-shielding analysis to obtain knowledge about the composite electromagnetic behavior. Differential scanning calorimetry (DSC) and thermogravimetric analysis (TGA) were performed to study the thermal decomposition behavior.

The novelty of this work is in the preparation of binary HAVOH/MXenes and ternary HAVOH/SWCNTs/MXenes composites, their complex study, which revealed remarkable EMI-shielding performance of hybrid composites, what is a promising basis for applications in this field.

## Methods

Multilayered, nondelaminated MXenes Ti_3_C_2_T_x_ were received as a water-based paste from Drexel University (Philadelphia, Pennsylvania, U.S.A.) with a concentration of 0.767 g of MXenes per 1 g of MXene paste. Highly amorphous poly(vinyl alcohol) (HAVOH) powder (commercialized under the trade name G-polymer, grade OKS-1089, Nippon Gohsei, Japan) was provided by Mitsubishi Chemicals. Single-walled carbon nanotubes (SWCNTs) (purity 80–93%) were purchased from Tuball™ (OCSiAl Europe S.a.r.l., Grand Duchy of Luxembourg).

**Figure 1:**
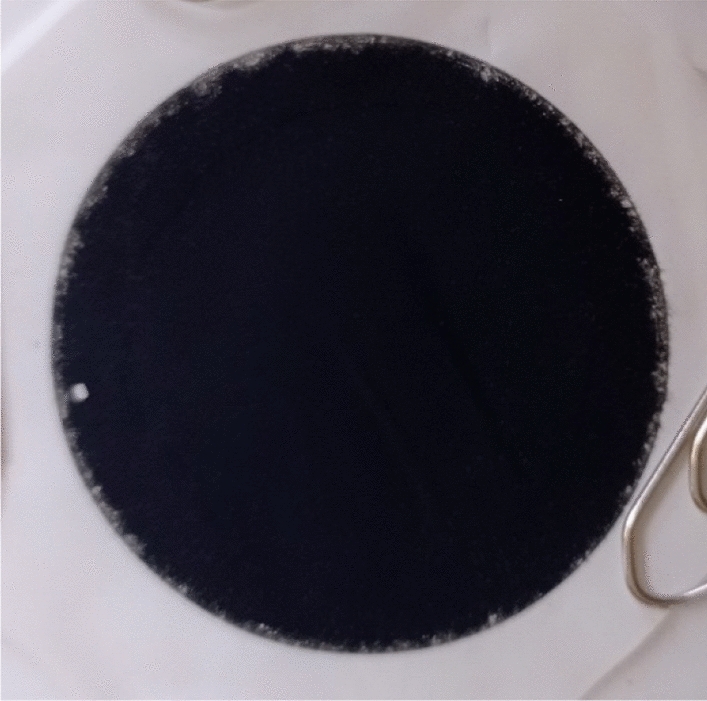
Free-standing film of MXenes prepared by VAF on a Celgard membrane (Ø = 4 cm, h = 18 μm).

To obtain a delaminated single-layered MXene solution, MXene paste was mixed with LiCl (1 g of Ti_3_C_2_T_x_:1 g of LiCl) in 20 ml of deionized (DI) water. The beaker with the mixture was placed into a water bath and placed on a magnetic stirrer. It was left at 35 °C and 150 rpm for stirring overnight. The next day, it was centrifuged at 3500 rpm until the supernatant became black. In further steps, the centrifugation cycle time was increased to 1 h. When the supernatant turned dark green, it contained delaminated single-layered MXene sheets, so it was the start of solution collection. When the solution started to appear light-green or transparent, centrifugation was stopped. To obtain the concentration of the prepared solution, using 20 mL of the solution and Celgard®3501 membrane, this portion of the solution was filtered with vacuum-assisted filtration (VAF), obtaining almost dried MXene powder as a film (Fig. 1). To remove residual water, the membrane with MXene film was put into a vacuum oven for drying at 45 °C until the next day. As a next step, the concentration of MXenes in the prepared single-layer solution was calculated. Concentrations were usually in the range of 0.1–0.5 mg/ml; therefore, the reconcentration process with a rotary vacuum evaporator was performed to increase the concentration.

**Figure 2:**
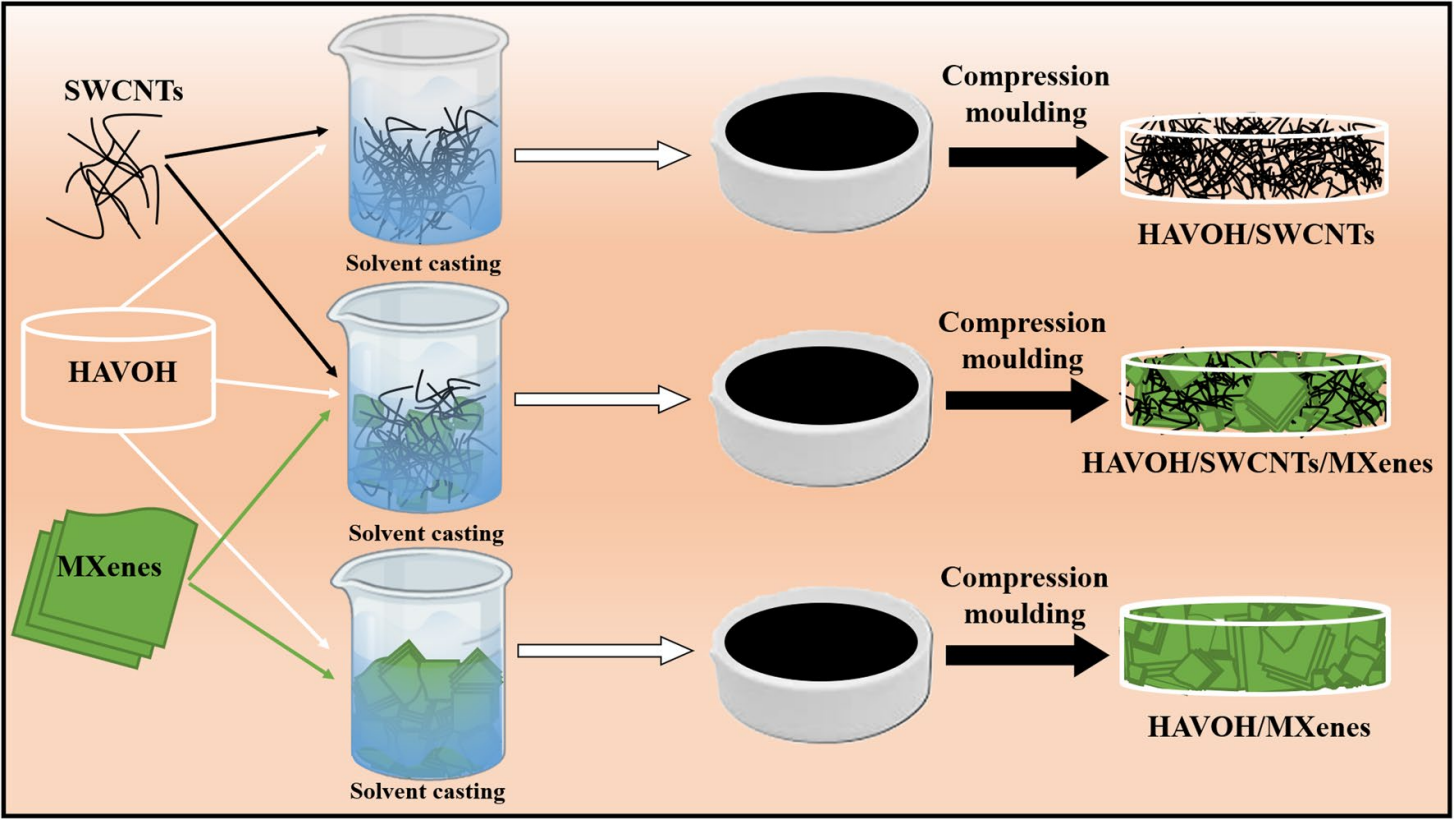
Scheme of preparation of three series of HAVOH polymeric nanocomposites.

All composites were prepared with solvent casting using water as a solvent. Table 1 summarizes the presented compositions and short names of the fabricated HAVOH-based polymeric nanocomposites, and Fig. 2 schematically shows the preparation processes.

**Table 1:** Composition of HAVOH-based specimens.

HAVOH (H), wt. %	SWCNTs, wt. %	MXenes (MX), wt. %	Short name
100	–	–	H
99.0	1.0	–	H/1 SWCNTs
97.0	3.0	–	H/3 SWCNTs
98.6	–	1.0	H/1 MX
97.0	–	3.0	H/3 MX
96.0	3.0	1.0	H/3 SWCNTs/1 MX
95.0	3.0	2.0	H/3 SWCNTs/2 MX
94.0	3.0	3.0	H/3 SWCNTs/3 MX

To prepare HAVOH/SWCNTs composite samples first, HAVOH powder was added to deionized (DI) water. Solutions containing 3.0 wt. % of polymer were prepared using a magnetic stirrer for 30 min at 50 °C, then the temperature was increased to 90 °C and left for further stirring for 30 min. Depending on the concentration of the filler in the sample, a calculated amount of SWCNTs was added to DI water and sonicated with an ultrasonic probe for 1 h (100% amplitude, cycle—1). Then, the mixture was added to HAVOH and left for mixing under continuous stirring for 30 min. In the next step, the HAVOH/SWCNTs mixture was mixed at 1200 rpm for an additional 3 h using a mechanical stirrer. Afterward, the mixture was poured out into a polytetrafluoroethylene (PTFE) Petri dish and dried at 60 °C under vacuum for total water evaporation, which took 5 days. This film was cut into small pieces for further processing with compression molding.

For preparation of HAVOH/MXenes composite samples the HAVOH dispersion preparation procedure was the same as described above. According to the desired filler load, a calculated amount of MXene solution was added to dissolved HAVOH and left for mixing for 30 min. In the next step, the beaker containing the HAVOH/MXene mixture was mechanically stirred at 1200 rpm for 3 h. Then, the mixture was poured out into a PTFE Petri dish dried at 60 °C under vacuum for 5 days for total water evaporation, and the obtained film was cut for compression molding.

To obtain HAVOH/SWCNTs/MXenes hybrid composites, the preparation procedures described above were combined. First, HAVOH was dissolved in water, and then SWCNTs and MXene suspensions were added and mixed on a magnetic stirrer. Afterward, the mixture was mechanically stirred at 1200 rpm for 3 h, poured into PTFE Petri dishes and processed as described above.

Circles of 0.3 mm thickness and 2.5 cm in diameter were prepared by compression molding of the prepared composites using a laboratory hydraulic press SRA 100 (Fontijne, Netherlands) at 2.4 MPa and at 220 °C for 10 min.

X-ray photoelectron spectroscopy (XPS) signals were recorded using a Thermo Scientific NEXSA G2 Surface Analysis System (Thermo Fisher Scientific, UK) equipped with a microfocused, monochromatic Al Kα X-ray source (1486.68 eV). An X-ray beam of 400 µm size was used. Spectra were acquired in the constant analyzer energy mode with a pass energy of 200 eV for the survey. Narrow regions were collected using a pass energy of 50 eV. Charge compensation was achieved with the system dual beam flood gun. Thermo Scientific Avantage software, version 6.6.0 (Thermo Fisher Scientific, UK), was used for digital acquisition and data processing. Spectral calibration was determined by using the automated calibration routine and the internal Au, Ag and Cu standards supplied with the K-Alpha system. Surface compositions (in atomic %) were determined by considering the integrated peak areas of the detected atoms and the respective sensitivity factors.

The morphology of the investigated samples was observed by a JEOL 7600F Schottky field emission scanning electron microscope (SEM FES) (Jeol Ltd., Tokyo, Japan). The nanocomposites with HAVOH as the matrix were investigated at an accelerating voltage of 5 kV in high vacuum. Before SEM analysis, samples were sputtered with a thin layer of gold by a Balzers SCD 040 coater (Balzers Union Limited, Balzers, Liechtenstein). AzTec software (Springfield, NJ, USA) was used to collect figures. For SEM, all samples were fractured in liquid nitrogen, and afterward, these cross-sectional areas were scanned.

Bright-field transmission electron microscopy (TEM) analysis was carried out by means of a FEI Tecnai G12 Spirit Twin (LaB6 source) equipped with a FEI Eagle 4 k CCD camera. The accelerating voltage was set at 120 kV. Before analysis, ultrathin sections of nanocomposite samples (nominal thickness 150 nm) were obtained at room temperature under dry conditions by using a Leica UC6 ultramicrotome and deposited on 400 mesh copper grids.

Broadband dielectric spectroscopy (BDS) measurements were performed by a Novocontrol Concept 40 with an Alpha dielectric spectrometer provided by Novocontrol Technologies GmbH (Germany) in the frequency range of 10^–1^ Hz to 10^6^ Hz. A BDS 1200 (supplied by Novocontrol Technologies) parallel-plate capacitor with two gold-plated electrodes was used as a test cell. AC parameters were measured after vacuum depositing gold electrodes (20 mm in diameter) on both sides of pelletized samples to ensure electrical contact. Frequency scans were conducted for each of the examined specimens. The diameter of the specimens was 40 mm. Thickness ranges were between 0.2 and 0.5 mm depending on the specimen. The system is fully automated, and WinDeta software was used for system control and data acquisition.

The EMI shielding effectiveness of the prepared nanocomposite sheets (length × width = 2.3 × 1 cm, thickness—1 mm) was studied with a vector network analyzer (Agilent N5230A) at 8.2–12.4 GHz frequency range (the so-called X-band) using a waveguide sample holder.

The electromagnetic interference shielding refers to the attenuation of the transmitting electromagnetic waves by the shielding material. A high value of electromagnetic interference (EMI) shielding effectiveness (SE) means less energy transmitted through the shielding material. For commercial applications, a shielding material which possesses the (SE) of 20 dB can block 99% of the incident electromagnetic waves. The electromagnetic shielding effectiveness (SE) can be expressed as the ration of transmitted power corresponding to the incident power of the EM wave, as:

1 $$SE\left(\mathrm{dB}\right)={\mathrm{SE}}_{R}+{\mathrm{SE}}_{A}+{\mathrm{SE}}_{M}=10\mathrm{log}\left(\frac{{P}_{T}}{{P}_{I}}\right)=20\mathrm{log}\left(\frac{{E}_{T}}{{E}_{I}}\right)=20\mathrm{log}\left(\frac{{H}_{T}}{{H}_{I}}\right) ,$$

where, P_T_ (E_T_ or H_T_) and P_I_ (E_I_ or H_I_) symbolized transmitted power and initial power (electric and magnetic field intensity) of EM wave respectively. Here, SE_R_ and SE_A_ are the shielding effectiveness because of reflection and absorption, respectively. SE_M_ is the shielding effectiveness due to multiple reflections inside the material, which can be negligible when SE_T_ > 10 dB. The total shielding efficiency (SE_T_) is given as Eq. (2):

2 $${\mathrm{SE}}_{T}\left(\mathrm{dB}\right)={\mathrm{SE}}_{R}+{\mathrm{SE}}_{A} .$$

The shielding effectiveness of the (magnetic/conductive) polymer composite filled e.g. with graphene oxide can be evaluated on the basis of scattering parameters (S_11_, S_12_, S_21_, S_22_) by following Eqs. (3), (4):

3 $${SE}_{R}=10{\mathrm{log}}_{10}\left(\frac{1}{1-R}\right)={10\mathrm{log}}_{10}\left(\frac{1}{1-{\left|{S}_{11}\right|}^{2}}\right)$$

4 $${SE}_{A}=10 {\mathrm{log}}_{10}\left(\frac{1-R}{T}\right)=10{\mathrm{log}}_{10}\left(\frac{1-{\left|{S}_{11}\right|}^{2}}{{\left|{S}_{21}\right|}^{2}}\right).$$

A two-port network analyser can be utilized to measure the scattering parameters (S_11_, S_12_, S_21_ and S_22_), which correlates with reflection (R) and transmission coefficients (T):

5 $$T={\left|{S}_{12}\right|}^{2}={\left|{S}_{21}\right|}^{2}$$

6 $$R={\left|{S}_{11}\right|}^{2}={\left|{S}_{22}\right|}^{2}.$$

The relationship between reflected (R), absorbed (A), and transmitted (T) portions of electromagnetic wave intensity follows:

7 $${R}^{2}+{A}^{2}+{T}^{2}=1$$

Using this equation, direct determination of R and T from measured values of S_11_ and S_21_, respectively, enables one to extract absorbed part (A) of electromagnetic wave intensity^11^.

Thermal characterization was performed using a differential scanning calorimeter (DSC-Q1000, TA, USA) in a flowing nitrogen atmosphere with a gas flow rate of 50 mL/min. Samples were preliminarily heated from 0 to 220 °C with a heating rate of 10 °C/min to erase their thermal history. Then, after an isotherm at 220 °C for 5 min, a cooling scan until 0 °C at 10 °C/min and a second heating scan from 0 to 220 °C at a rate of 10 °C/min were performed. Thermal parameters (glass transition temperature T_g_, melting temperature T_m_, and melting enthalpy ΔH_m_) on the second heating scan were calculated.

Thermogravimetric analysis (TGA) measurements were carried out on a TGA Q500 thermogravimetric analyzer (TA Instruments, USA). For the actual analysis, a small amount of sample (on the order of tens of mg) was prepared and transferred into a platinum pan. Measurements were carried out at a heating rate of 10 °C/min from laboratory temperature to 900 °C in an air atmosphere.

### Consent to participate

All authors contributed to the research presented in the paper and approved their participation.

## Results and discussion

**Figure 3:**
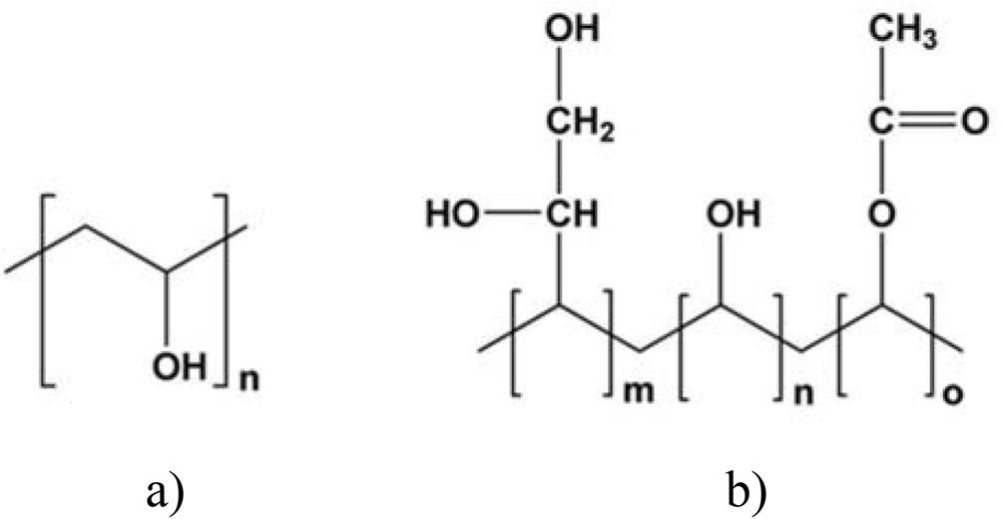
Chemical structure of (**a**) PVOH and (**b**) HAVOH.

Highly amorphous poly(vinyl alcohol) is a biodegradable polymer based on poly(vinyl alcohol) modified with diol monomers (Fig. 3), which is also water soluble. There are only a few water-soluble polymers. This property was favorable for our work on the fabrication of HAVOH-based composites with MXenes because MXenes were received as a water-based paste, and their further processing, *e.g.,* delamination, was also performed in water. In this study, we worked with nonoxidized MXene paste, which is described in detail here (labelled “MX2” in Ref.^35^). Delaminated MXene showed no oxidation (only 2.9 at. % of Ti^4+^ signal ca. 458.5 eV) and was very clean with a low amount of sp^3^ carbon of 6.1 at. % (Fig. S1, Table S1). Only a small amount of aluminum remained from the MAX phase in the structure (Table S1). The hydrophilic character of this type of MXene was discussed and proven in Ref.^36^. Thus, it was not necessary to transfer these 2D fillers from water to another solvent, which could cause additional difficulties, such as lower yield due to losses during the transfer process, agglomeration and sedimentation. As a 1D nanofiller, single-walled carbon nanotubes (SWCNTs) were used. The main reason is that SWCNTs have higher electrical conductivity than MWCNTs^37,38^. SWCNTs are hydrophobic, which is also evident from the surface composition determined by XPS, where approximately 80.9 at. % sp^2^ carbon is on the surface and only approximately 1.7 at. % oxygen provides hydrophilic character (see Fig. S2 and Table S2). Therefore, it was challenging to prepare homogeneous composites. We prepared and characterized binary and ternary composites with the HAVOH matrix and 1D SWCNTs and 2D MXenes as fillers (Table 1).

Study of the morphology and intrinsic structure are very important characteristics for further interpretation of the material properties. The orientation of the fillers, their dispersion, presence of agglomerates, incorporation into the polymer matrix, interaction between the matrix and the filler with other fillers or additional helping components, *e.g.,* copolymers, surfactants, compatibilizers, etc., affect the properties of the final material. Poor dispersion of fillers, the presence of agglomerates, and incompletely evaporated solvent residues can worsen the electrical and mechanical properties of polymeric nanocomposites.

**Figure 4:**
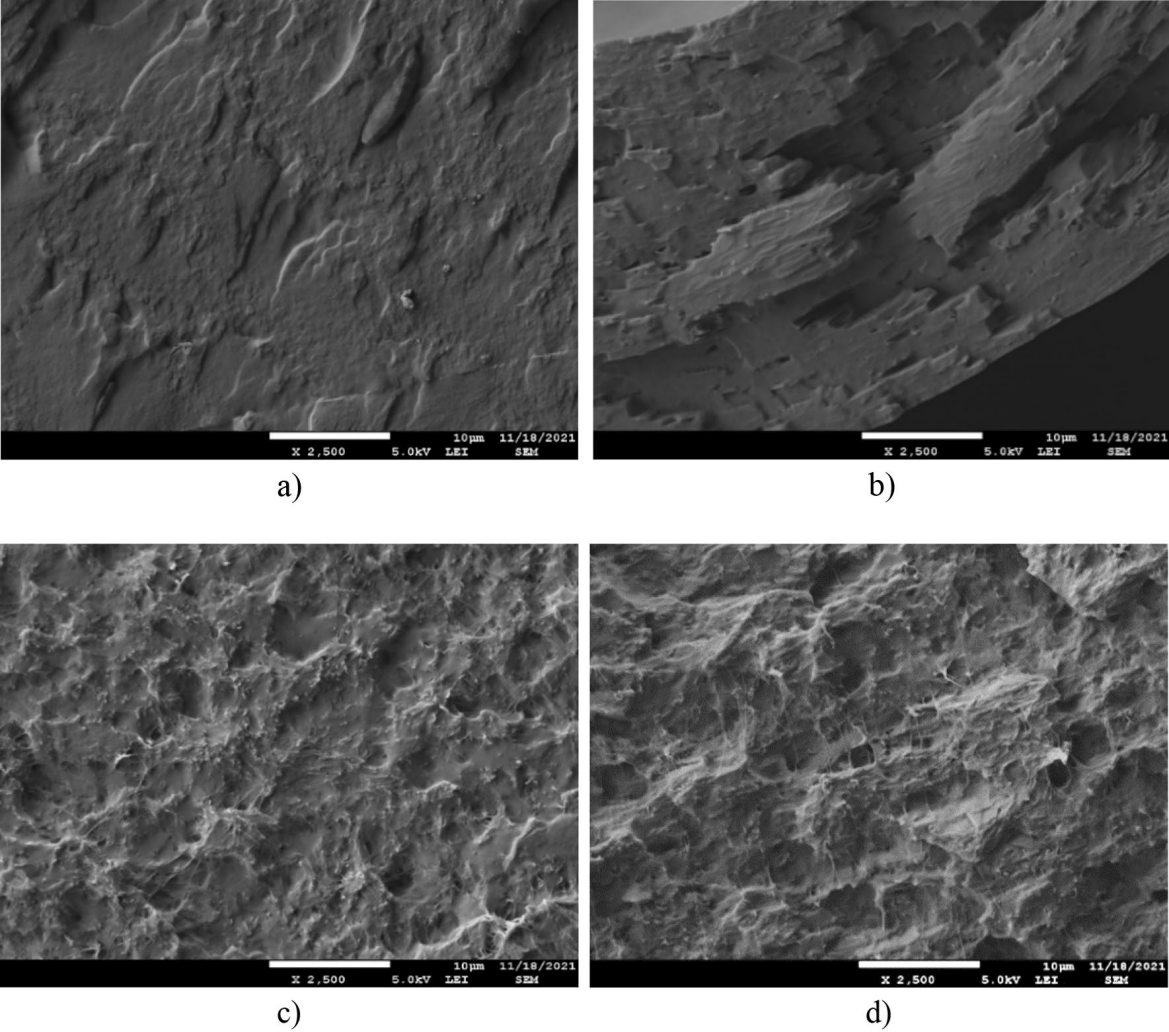
SEM images of (**a**) neat H, (**b**) H/1 MX, (**c**) H/3 SWCNTs; and (**d**) H/3 SWCNTs/1 MX.

Study of the morphology and structure of HAVOH-based composite samples was performed with SEM and TEM. In Fig. 4, SEM micrographs of the pure HAVOH matrix (Fig. 4a), binary composites HAVOH/1.0 wt. % MXenes (Fig. 4b), HAVOH/3.0 wt. % SWCNTs (Fig. 4c), and the ternary hybrid HAVOH/3.0 wt. % SWCNTs/1.0 wt. % MXenes (Fig. 4d) are presented. All composites reveal a homogeneous morphology. When 2D MXene nanofiller was added to HAVOH, the formation of lamellar filler structures parallel to the sample surface was observed. This can be attributed to the MXene layered structure.

For the binary HAVOH/3.0 wt. % SWCNTs (H/3 SWCNTs) sample (Fig. 4c), instead of ordered lamellar structures, well dispersed SWCNTs without large agglomerates are observed. As a result of mixing of 1D and 2D nanofillers, such as for the sample HAVOH/3.0 wt. % SWCNTs/1.0 wt. % MXenes (H/3 SWCNTs/1 MX) (Fig. 4d), a combination of individual structures of binary composites with utilized fillers is observed. In particular, either lamella structures typical of MXenes or well-dispersed SWCNTs that are able to connect MXene lamellae are observed. Thus, improving the electrical conductivity of the samples by the creation of continuous conductive pathways is present.

**Figure 5:**
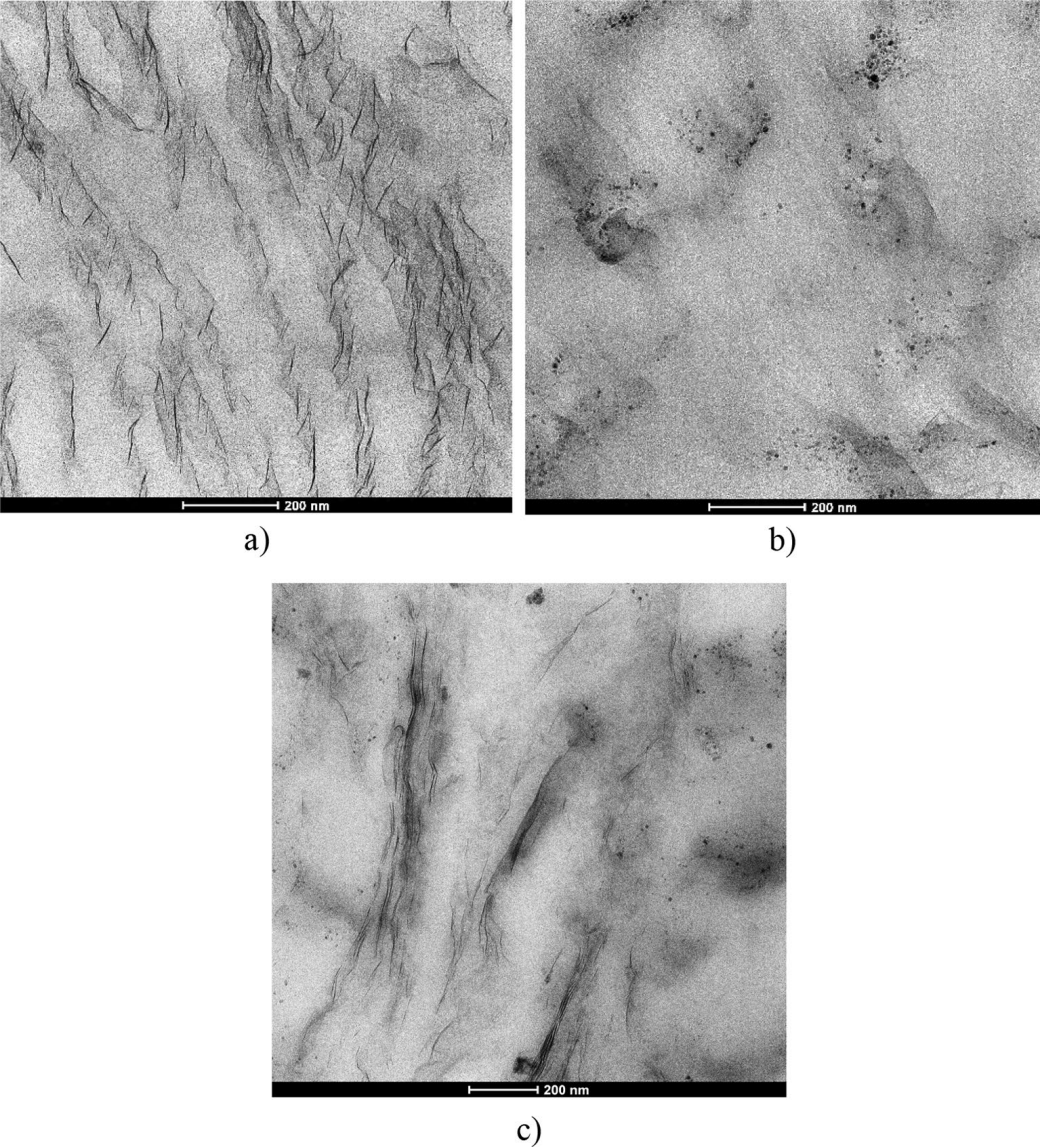
TEM images of (**a**) H/3 MX; (**b**) H/3 SWCNTs; and (**c**) H/3 SWCNTs/3 MX.

Further insights into the dispersion and orientation of the filler within the HAVOH matrix were provided with TEM analysis. TEM micrographs of the binary and ternary composites are shown in Fig. 5 and additional photos can be found in SI (Fig. S3). Figure 5a shows a bright-field TEM micrograph of H/3 MX composite sample with 3.0 wt. % of 2D MXenes, with a well-demonstrated layered structure attributed to the alignment of MXene lamellae to the surface of the film, confirming what was already evidenced by SEM analysis. MXene lamellae are tightly packed with each other with regular morphology.

For the HAVOH/3.0 wt.% SWCNTs binary composite (Fig. 5b), CNT bundles can be clearly observed. They have a loose structure, which is evidenced in the micrograph as gray/black halos, because single nanotubes cannot be resolved due to their very low diameter. When the two fillers were combined together, a continuous net of hybrid MXenes/SWCNTs was observed (Fig. 5c). SWCNTs, in this case also evidenced as low-contrast bundles in comparison to high-atomic-number MXenes, contribute to the connection of the MXene lamellae, which clearly indicates the formation of 3D hybrid conductive pathways within the nanocomposite structure that are responsible for the improved electrical conductivity. For this sample, the highly regular spatial arrangement of MXene lamellae evidenced for the binary HAVOH/MXene composite is partially lost as the arrangement of the lamellae is perturbed and hindered by the copresence of SWCNTs.

**Figure 6:**
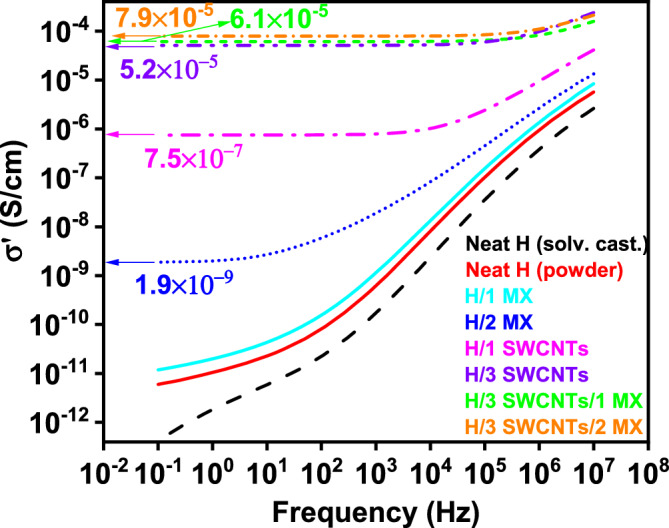
Dependency of real part of HAVOH/SWCNTs, HAVOH/MXenes, and HAVOH/SWCNTs/MXenes composite samples conductivities on frequency.

HAVOH-based samples were analyzed with BDS to study their electrical properties. Figure 6 shows a plot of the measured conductivities of the binary and ternary HAVOH composites. The neat matrix with a ${\sigma }_{DC}{\prime}$ conductivity value of 6 × 10^–12^ S/cm had a typical insulator response. For the composite with 2.0 wt. % MX, the conductivity increased to approximately 2 × 10^–9^ S/cm; beyond a critical frequency (fc), a power law followed, whereas for f < f_c_, σ′ exhibited a plateau, corresponding to DC conductivity (${\sigma }_{DC}{\prime}$)^39^. The addition of 2.0 wt. % MXenes to HAVOH slightly increases ${\sigma }_{DC}{\prime}$ conductivity, reaching 2 × 10^–9^ S/cm, while the composite sample with 1.0 wt. % of this filler has a similar curve shape (similar f_c_) and very close values to the neat matrix. The specimen with 1.0 wt. % of SWCNTs is on the same order range with a conductivity of 7.5 × 10^–7^ S/cm. Hybrid ternary composites, HAVOH/3.0 wt. % SWCNTs/1.0 wt. % MXenes and HAVOH/3.0 wt. % SWCNTs/2.0 wt. % MXenes, together with binary composite HAVOH/3.0 wt. % SWCNTs in between, are reaching more than two times better conductivity compared to the HAVOH matrix alone. Their ${\sigma }_{DC}{\prime}$ conductivity values, 5.2 × 10^–5^, 6.1 × 10^–5^ and 7.9 × 10^–5^ S/cm, respectively, are the highest among all fabricated HAVOH samples.

The percolation threshold for HAVOH binary composites was calculated using the equation and scaling law:

8 $${\sigma }_{DC}\approx {\left(p-{p}_{c}\right)}^{t},$$

where ${\sigma }_{DC}$ is the DC conductivity, *p*_*c*_ is the percolation threshold, *p* is the volume fraction of the filler, and *t* is the exponent characterizing the dimensionality of the investigated conductive system. In HAVOH/SWCNTs, *p*_*c*_ is 0.7 vol. %, which corresponds to 1.0 wt. %. For samples with MXenes filler, *p*_*c*_ was 0.56 vol. %, which in weight percentage is equal to 1.4 wt. %. As shown in this plot, the electrical conductivity of HAVOH-based composites did not exceed 8 × 10^–5^ S/cm. This is just one order of magnitude from values of 1.2×10-4 S/cm for HAVOH/6.0 wt. % MWCNT presented by Santillo et al.^5^. So, with slightly lower filler content 4 (3 wt.% SWCNTs + 1 wt.% MX) and 5 (3 wt.% SWCNTs + 2 wt.% MX) wt. % we achieved a very good conductive network penetrated into the HAVOH matrix without using any additional surfactant, as was the case of Santillo et al^5^.

**Figure 7:**
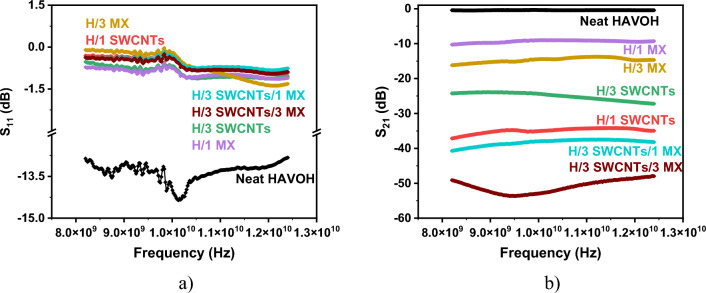
EMI-shielding efficiency expressed (**a**) by S_11_ parameter of the samples, and (**b**) by S_21_ parameter of the samples.

The EMI shielding properties for the neat HAVOH matrix as well as for nanocomposites with SWCNTs and MXene fillers in the frequency range from 8.2 to 12.4 GHz (X-band) were first determined and expressed by the S_21_ parameter. According to Fig. 7, the HAVOH matrix’s transparency in a given region is approximately 92%, i.e., its shielding efficiency in a composite without nanoparticles is approximately S_21_ = -0.45 dB, which is negligible. Parameter S_11_ for all composites indicates the high conductivity of the samples, and S_11_ approaches zero. The shielding efficiency S_21_ for composites with 1.0 wt. % SWCNTs exhibit increasing shielding (− 35 dB), while the sample with 3 wt. % of SWCNTs exhibits worse shielding, namely, − 25 dB. This is possibly caused by the poor quality of the sample, which is macroscopically irregular, with some holes and defects due to the high amount of SWCNTs. Shielding of samples with 1.0 and 3.0 wt. % of MXenes exhibits good shielding (− 10 and − 15 dB). Better results are obtained for samples containing both fillers (SWCNTs and MXenes), which exhibit great shielding of − 40 dB (HAVOH/3.0 wt. % SWCNTs/1.0 wt. % MXenes) and − 55 dB (HAVOH/3.0 wt. % SWCNTs/3.0 wt. % MXenes), which is much higher than the sample with 3.0 wt. % SWCNTs. This confirms that MXenes help to form a compact conductive network together with the SWCNTs and improve their EMI-shielding.

The morphology study showed that MXene has a lamellar structure and the well-dispersed SWCNTs formed a continuous conductive path between the lamellae. Thus, improving the electrical conductivity in composites HAVOH/ SWCNTs/MXenes is proof of that. Actually, a material absorbs EM waves and converts them into heat, and this conversion ability is determined by the conduction loss and the polarization loss. Polarization loss has an important contribution to the absorption of EM waves. In fact, the defects formed during the etching process as was introduced by Peng He et al.^40^ lead to the asymmetry in the spatial distribution of electrons to form dipole moments. Moreover, terminal atoms attached on the surface of MXenes (Fig. S1) lead to the asymmetric distribution of charge density, which in-turn causes the formation of dipoles. Under an alternating electromagnetic field, these dipoles will break loose and rotate directionally. When their rotation cannot keep up with the change in EM field, polarization relaxation occurs with EM energy loss. Recent researchers have mainly focused on the EMI shielding performance of Ti_3_C_2_T_x_ MXene films^31^. However, EM waves are mostly reflected by high conductivity in the Ti_3_C_2_T_x_ film and this behavior is the same as a metal shielding the EM wave. Only a small amount of EM waves can enter the body of films and be absorbed. Compared with the MXene films that mainly use high conductivity to reflect EM waves, the composites made by dispersing the SWCNTs and MXene into the HAVOH matrix can reduce the conductivity and absorb more EM waves by polarization loss. This means that more EM waves can be attenuated so that secondary reflections will be effectively reduced, demonstrating their environmentally friendly performance.

**Figure 8:**
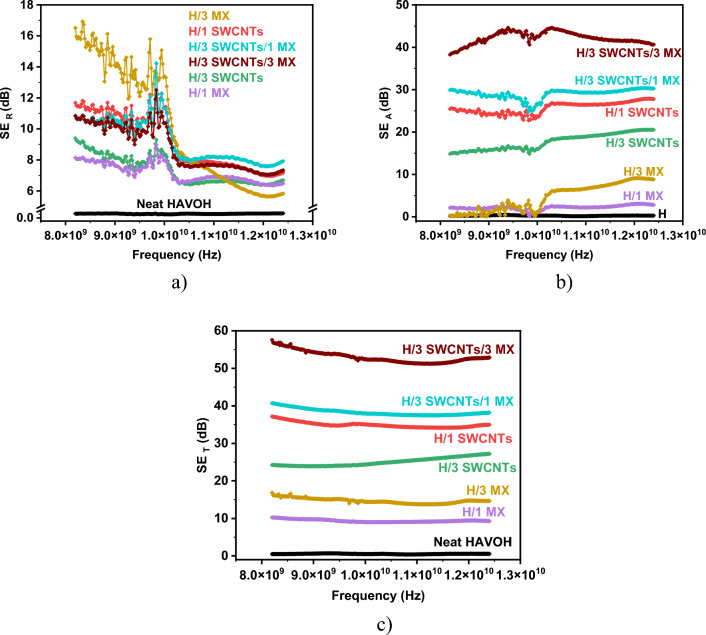
Split of (**a**) reflection part; (**b**) absorption part, and (**c**) total shielding effectiveness SE_T_.

Figure 8 shows the split of total shielding into absorption and reflection parts and provides another view of the shielding efficiency of HAVOH/SWCNTs/MXene morphologies. The reflectivity of all composites is quite similar, with values in the range of 7–15 dB. More interesting is the absorption of the samples, which show large differences among the samples. The samples filled with MXenes absorb much less than the samples filled with SWCNTs, which absorb up to 45 dB. The contribution of reflection and absorption can also be viewed in Table 3 and Fig. S4. Visualizing the proportion between the three components in “RAT analysis” (analysis of reflection, absorption, and transmission capability of each sample) clearly indicates higher absorption percentage of samples with MXene/SWCNTs hybrid structure.

**Table 3:** Reflection, transmission and absorption values at 10 and 11 GHz.

№	Name of the sample	10 GHz			11 GHz		
		R (%)	T (%)	A (%)	R (%)	T (%)	A (%)
1	Neat HAVOH	3.98	92.1	3.94	4.8	91.1	4.3
2	H/1 SWCNTs	92.8	0.017	7.2	83.5	0.04	16.4
3	H/3 SWCNTs	85.4	0.06	14.2	78.2	0.3	21.5
4	H/1 MX	84.0	12.5	3.5	79.7	12.3	8.1
5	H/3 MX	95.4	3.6	1.00	80.7	4.2	15.1
6	H/3 SWCNTs/1 MX	92.6	0.0016	7.4	84.9	0.017	15.1
7	H/3 SWCNTs/3 MX	90.8	0.005	9.2	82.95	0.009	17.0

*R* reflection,*T* transmission, *A* absorption.

The results obtained in this work were compared with other reported values of the shielding effectiveness of MXene composites. Miao et al.^41^ obtained 54.7 dB of RL_min_ (extreme reflection loss) for the MoS_2_/TiO_2_/Ti_2_CT_x_ sample. This value is the closest to that reported here; however, the sample thickness is three times higher than our HAVOH/3.0 wt. % SWCNTs/3.0 wt. % MXenes. Additionally, the filler ratio that has to be used is high, reaching 70 wt. %, which is almost 12 times more than for our specimen with a total filler load of 6 wt.%. Gao et al.^42^ also received high SE values. Prepared composite with thermoplastic polyurethane as a matrix and MXenes filler showed 50.7 dB, but high amount of filler load such as 28.6 wt. % had to be admixed.

HAVOH composites showed great shielding effectiveness. When comparing the neat polymer matrix and the composite sample that showed the best results, namely, HAVOH/3.0 wt. % SWCNTs/3.0 wt. % MXenes, the difference is more than 100 times. This indicates that utilization of both fillers in relatively small amounts and a very good synergistic effect greatly increases reflection and absorption and decreases transmission of electromagnetic interference, thus creating a great barrier. These results are promising for further development of EMI-shielding films or devices.

To study thermal behavior of the specimens differential scanning calorimetry and thermogravimetric analyses were used. DSC thermograms of pristine HAVOH and HAVOH-based composite samples, recorded during cooling from the melt and successive heating of the melt-crystallized samples, are shown in Fig. 6. Values of glass transition temperatures (T_g_), melting temperatures (T_m_) and melting enthalpy (ΔH_m_) are summarized in Table 2.

**Table 2:** Glass transition temperatures (T_g_), melting temperatures (T_m_) and melting enthalpy (ΔH_m_) of HAVOH composites.

Sample	T_g_ (°C)	T_m_ (°C)	ΔH_m_ (J/g)
H	75.5	199.2	34.2
H/1 SWCNTs	79.9	183.9	30.4
H/3 SWCNTs	79.4	198.7	37.7
H/1 MX	75.7	197.5	39.4
H/3 MX	73.9	192.1	34.9
H/3 SWCNTs/1 MX	81.2	191.3	30.2
H/3 SWCNTs/3 MX	76.3	187.8	32.1

**Figure 9:**
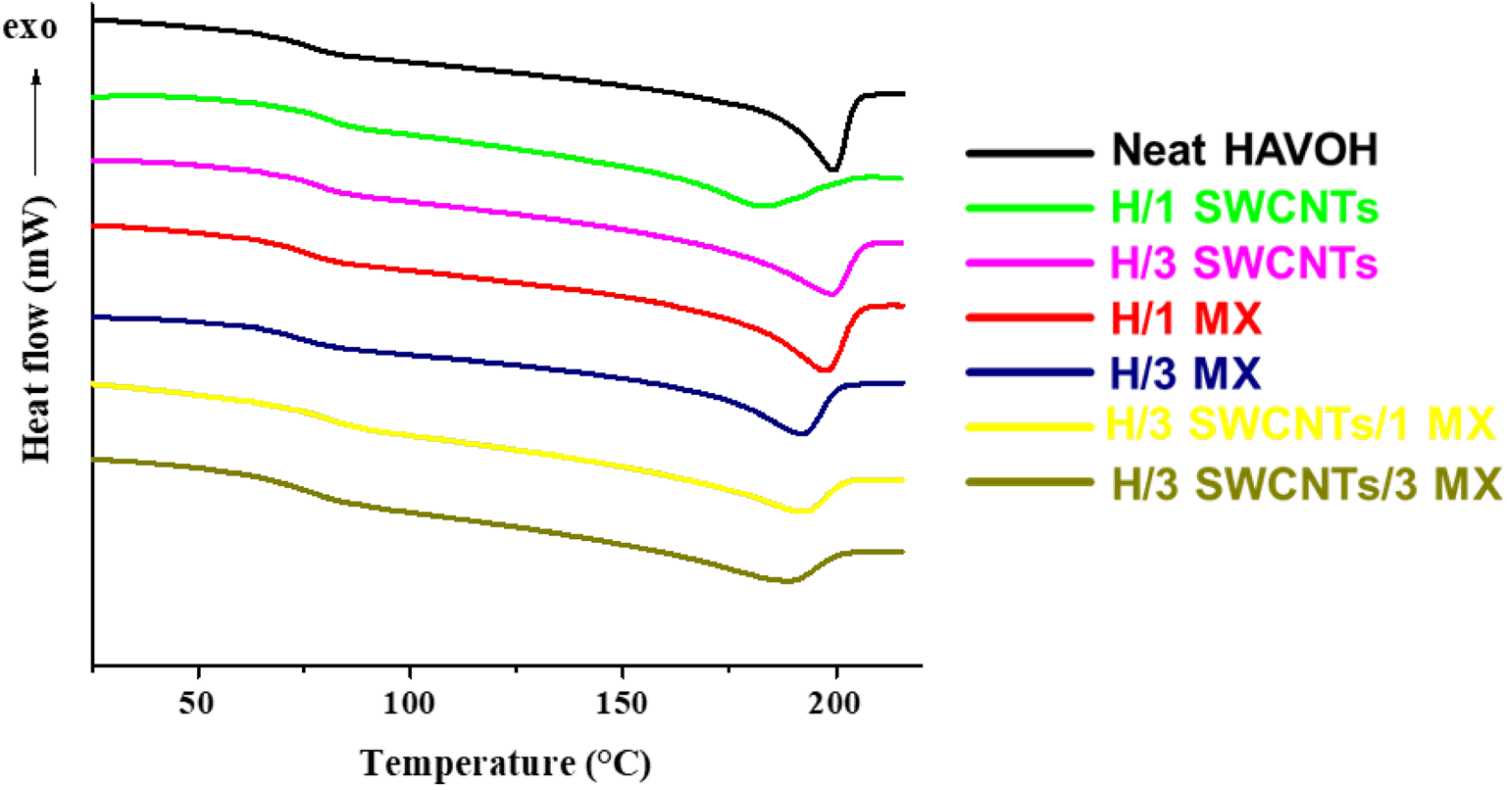
DSC thermograms of HAVOH and related composites.

As shown, the DSC heating curve of pristine HAVOH, crystallized from the melt at 10 C/min, displays a T_g_ of 75.5 °C, a T_m_ of 199.2 °C and a ΔH_m_ of 34.2 J/g (Fig. 9 and Table 2). For binary composites containing SWCNTs, an increase in T_g_ is observed, almost irrespective of the SWCNTs amount up to 79.9 °C, whereas for the melting temperature, a decrease in T_m_ is observed at 1.0 wt. % SWCNTs loading, while at 3.0 wt. % SWCNTs, the T_m_ value is quite similar to that recorded for neat HAVOH. The different effect on melting, induced by the amount of SWCNTs, is confirmed by comparing the melting enthalpy values, as the sample containing 1.0 wt. % SWCNTs shows a significant decrease in ΔH_m_, while at 3.0 wt. % of SWCNTs loading ΔH_m_ increases in comparison to neat HAVOH. For binary composites containing MXenes, at either 1.0 wt. % and 3.0 wt. % MXene loading, a decrease in the T_g_ is observed in comparison to composites containing SWCNTs. T_g_ values of HAVOH/1.0 wt. % MXenes and HAVOH/3.0 wt. % MXenes are similar to that recorded for neat HAVOH. For the melting temperature, a progressive decrease in T_m_ is recorded with increasing MXene amount, while for the melting enthalpy, an increase in ΔH_m_ is recorded at 1.0 wt. % MXene loading, while at 3.0 wt. % MXene loading ΔH_m_ returns very close to that recorded for pristine HAVOH, indicating that lower amounts of MXenes can induce more pronounced crystallization. Finally, for ternary samples containing 3.0 wt. % SWCNTs and different amounts of MXenes, it is observed that at 1.0 wt. % MXenes loading, the amount of SWCNTs is predominant in the T_g_ values, with the T_g_ of HAVOH/3.0 wt. % SWCNTs/1.0 wt. % MXenes similar to that recorded for the binary composite HAVOH/3.0 wt. % SWCNTs while increasing the amount of MXenes to 3.0 wt. %, T_g_ decreases to a value close to that recorded for neat HAVOH, with a trend similar to that observed for binary composites containing only MXenes. Moreover, for the melting temperature, a decrease in the T_m_ values is recorded for both ternary samples in comparison to neat HAVOH, and the T_m_ values of HAVOH composites containing 3.0 wt. % SWCNTs and 1.0 or 3.0 wt. % of MXenes are lower in comparison to those recorded for the corresponding binary composites containing only MXenes. Finally, for melting enthalpy values, both samples show a reduced crystallinity in comparison to neat HAVOH and all the binary composites, with ΔH_m_ values of 30.2 and 32.1 J/g at 1.0 wt. % and 3.0 wt. % MXene loading, respectively. Thus, the overall DSC results show a complex effect of the SWCNTs and MXene amounts on the thermal parameters of the HAVOH phase. In particular, for binary composites containing MXenes, at low MXenes amounts (1.0 wt. %) there is a negligible effect of the filler on T_g_ and T_m,_ whereas the filler induces an increase of the polymer phase crystallinity, possibly acting as a nucleating agent. Instead, at higher MXenes amounts (3.0 wt. %), a decrease of T_g_ and T_m_ is recorded, and the crystallinity becomes similar to that showed by neat HAVOH, possibly indicating that large amounts of MXenes hinder the polymer crystallinity. As concerning ternary composites containing both SWCNTs and MXenes, an increase in T_g_ is observed only for the HAVOH/3.0 wt. % SWCNTs/1.0 wt. % MXenes, whereas both HAVOH/3.0 wt. % SWCNTs/1.0 wt. % MXenes and HAVOH/3.0 wt. % SWCNTs/3.0 wt. % MXenes show a decrease in the melting temperature and melting enthalpy in comparison to neat HAVOH, showing that the percolative hybrid network observed for the ternary composites by SEM/TEM analysis hinders the crystallization of the polymer phase.

**Figure 10:**
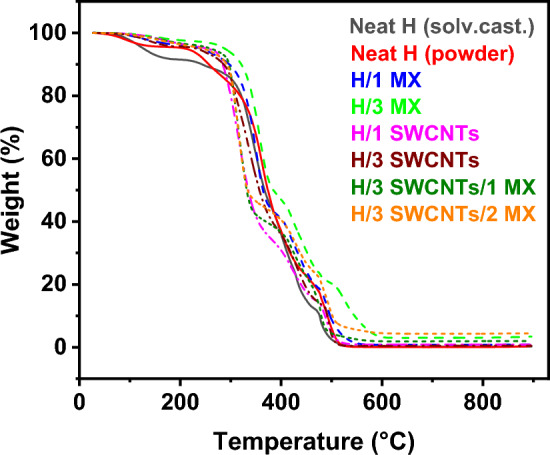
Plot of weight-loss temperature dependency of all HAVOH-based composites.

**Figure 11:**
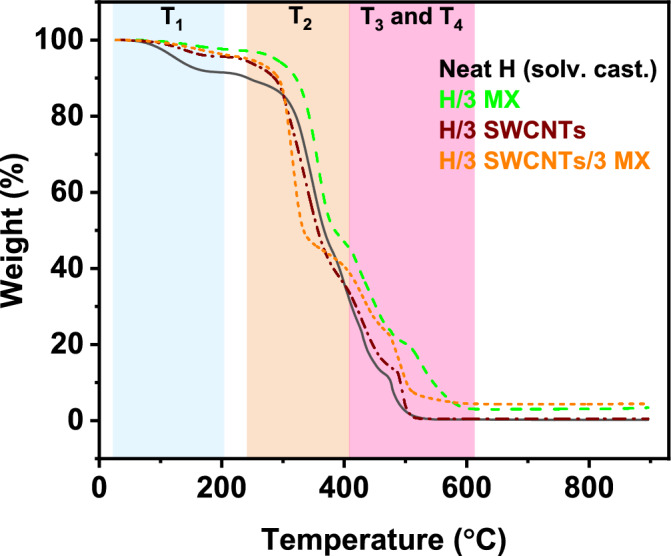
Temperature ranges of T_1_-T_4_°C degradation steps.

The TGA curves of neat HAVOH and binary and ternary composites are reported in Fig. 10. They reveal different mass loss zones that are better highlighted in the corresponding DTA (differential thermal analysis) curves (Fig. S5). All samples show four thermal degradation steps (stages), which are presented as T_1_, T_2_, T_3_, and T_4_ °C in Fig. 11. The T_1_ region, the first mass loss zone, which is between 25 and 200 °C (Fig. S5), corresponds to the loss of moisture and chemically and physically bonded water. The more hydrophilic it is, the bigger will be the peak and the lower will be T_0_ (beginning of the water loss stage). The T_2_ region in the temperature range from 250 to 400 °C corresponds to the decomposition of side chains of HAVOH (Fig. 3), and the next two degradation steps T_3_ and T_4_ (Fig. S6a)—from 400 °C and higher—are related to further decomposition of ethylene segments with conversion into carbon chains^5^.

There is some difference in the behavior of the neat HAVOH matrix and HAVOH powder. HAVOH powder was used for measurements in its initial form. Neat HAVOH matrix was prepared by dissolving of HAVOH powder in water under temperature and continuous stirring and then poured into a Teflon petri dish and dried. The first degradation step, presented in magnified resolution in Fig. S6b, shows that the specimen prepared with solvent casting (black solid line) has greater mass loss than the powder (red solid line), as the latter does not have water in general, and due to residuals of water nonevaporated during the vacuum-drying process. In the first step, T_1_ showed a difference between the two pure HAVOH matrices. After solvent casting, there is clearly more structural water in the material, resulting in higher weight loss in the first stage up to 200 °C (Fig. S5). The main peak of decomposition of the T_2_ stage is slightly shifted from 362 °C for HAVOH powder to 347 °C after solvent casting HAVOH. This might indicate more accessible side chains for faster degradation, but the residue at 395 °C is the same (Table S3). The reason for more accessible side chains could be either smaller crystallites or generally lower crystallinity.

The effect of nanofillers is very complex, as described above. Both types of nanomaterials contain residues of metals (Al in the case of MX and Fe in the case of SWCNTs, see Tables S1 and S2), which could accelerate the degradation. Samples with lower crystallinity (H/1 SWCNTs, H/3 SWCNTs/1 MX, H/3 SWCNTs/3 MX, see Table 2) show a strong shift of the main peak of the T_2_ region (from 347 °C for HAVOH down to 314–316 °C for these composites) related to the degradation of side chains. This confirms that the lower crystallinity means more accessible side chains, which together with some catalytic activity of nanofillers leads to faster degradation. MX samples showed slightly higher residues at 395 °C. There are several explanations for the stabilizing effect of nanofillers, such as heat dissipation, radical scavenging, and physical/chemical adsorption of the decomposition products^43^. In the case of MX, the stabilizing effect might be mainly due to the physical/chemical adsorption of the decomposition products, which is typical for 2D layered nanofillers such as MX. The residues of MX samples at 895 °C represent nondegraded nanofillers and roughly follow the initial concentrations. MXenes can even gain weight as they oxidize during thermal treatment in air^44^, while SWCNTs almost completely degrade^45^.

## Conclusions

Based on the HAVOH matrix, three series of composites were prepared: binary HAVOH/SWCNTs, HAVOH/MXenes, and ternary HAVOH/SWCNTs/MXenes. The highest electrical conductivity of 7.9 × 10^–5^ S/cm was achieved for the hybrid ternary composite HAVOH/3.0 wt. % SWCNTs/2.0 wt. % MXenes. SEM and TEM studies of the morphology and structure of HAVOH-based polymeric nanocomposites revealed homogeneously dispersed SWCNTs and MXenes within the matrix, and large agglomerates or clusters of nanofillers were not detected. These results confirmed the formation of a good conductive network through the polymeric matrix, which is based on the connections of 2D MXene nanosheets with carbon nanotubes. An EMI-shielding study confirmed the great shielding effectiveness (SE) of HAVOH-based binary and ternary composites. Hybrid composite fabricated with both MXenes and SWCNTs added in equal amounts of 3.0 wt. %, revealed a value as high as 55 dB, which is 122 times higher than that of the neat polymeric matrix. Thermogravimetric analysis was performed to study the influence of fillers on the thermal stability of HAVOH and composites. Slightly higher decomposition temperatures for both binary and ternary nanocomposites with MXenes and carbon nanotubes were revealed.

### Supplementary Information

Supplementary Information.

## Data Availability

All data are available in the paper and in the supporting information.

## References

[1] Shibutani M, Kanda T, Yamamoto T, Tokumitsu K (2017). Characteristics of the amorphous polyvinyl alcohol resin derivative having side chain 1,2-diol. J. Soc. Mater. Sci..

[2] Russo P (2015). Structure and physical properties of high amorphous polyvinyl alcohol/clay composites. AIP Conf. Proc..

[3] Donato KZ (2017). High amorphous vinyl alcohol-silica bionanocomposites: Tuning interface interactions with ionic liquids. ACS Sustain. Chem. Eng..

[4] Yan N (2015). Gas-barrier hybrid coatings by the assembly of novel poly (vinyl alcohol) and reduced graphene oxide layers through cross-linking with zirconium adducts. ACS Appl. Mater. Interfaces.

[5] Santillo C (2021). Tuning the structural and functional properties of HAVOH-based composites via ionic liquid tailoring of MWCNTs distribution. Compos. Sci. Technol..

[6] Guadagno L (2021). Flexible eco-friendly multilayer film heaters. Composites B.

[7] Wang Y-L (2020). Effect of mercapto-silanes on the functional properties of highly amorphous vinyl alcohol composites with reduced graphene oxide and cellulose nanocrystals. Compos. Sci. Technol..

[8] Guadagno L (2021). Eco-friendly polymer nanocomposites designed for self-healing applications. Polymer.

[9] Stepura, A. *et al*. Polymeric nanocomposites with hybrid nanofillers. In EPF European Polymer Congress: 26 June - 1 July 2022: book of abstracts. 1. - Prague, Czech Republic: AMCA, spol. s.r.o., 2022, p. 317. ISBN 978–80–88214–33–5.

[10] Omastová, M. *et al*. Polymeric nanocomposites with hybrid two- and one-dimensional fillers. In The 6th International conference on nanomaterials: Fundamentals and applications: Book of abstracts. Košice, 16.-19.10.2022. Edited by Jana Shepa; reviewed by Erika Múdra, Ivan Shepa. - Košice: Prírodovedecká fakulta UPJŠ, 2022, pp. 106–107.

[11] Yadav RS, Kuřitka Ivo, Vilčáková J (2020). Advanced spinel ferrite nanocomposites for electromagnetic interference shielding applications. Technol. Eng. Mater. Sci..

[12] Cao M (2018). Graphene nanohybrids: Excellent electromagnetic properties for the absorbing and shielding of electromagnetic waves. J. Mater. Chem. C.

[13] Naguib M (2011). Two-dimensional nanocrystals produced by exfoliation of Ti_3_AlC_2_. Adv. Mater..

[14] Naguib M, Mochalin VN, Barsoum MW, Gogotsi Yu (2014). 25th anniversary article: MXenes: A new family of two-dimensional materials. Adv. Mater..

[15] Khaledialidusti R, Anasori B, Barnoush A (2020). Temperature-dependent mechanical properties of Ti_n+1_C_n_O_2_ (n = 1, 2) MXene monolayers: A first-principles study. PCCP.

[16] Liu R, Li W (2018). High-thermal-stability and high-thermal-conductivity Ti_3_C_2_T_x_ MXene/poly(vinyl alcohol) (PVA) composites. ACS Omega.

[17] Zeraati ASh (2021). Improved synthesis of Ti_3_C_2_T_x_ MXenes resulting in exceptional electrical conductivity, high synthesis yield, and enhanced capacitance. Nanoscale.

[18] Balci E, Akkus UO, Berber S (2017). Band gap modification in doped MXene: Sc_2_CF_2_. J. Mater. Chem. C.

[19] Han M (2020). Tailoring electronic and optical properties of MXenes through forming solid solutions. J. Am. Chem. Soc..

[20] Iqbal A, Sambyal P, Koo CM (2020). 2D MXenes for electromagnetic shielding: A review. Adv. Func. Mater..

[21] Zhu Q, Li J, Simon P, Xu B (2021). Two-dimensional MXenes for electrochemical capacitor applications: Progress, challenges and perspectives. Energy Storage Mater..

[22] Zhang T-Y (2022). High-efficiency ultraviolet shielding and high transparency of Ti_3_C_2_T_x_ MXene/poly(vinyl alcohol) nanocomposite films. Compos. Commun..

[23] Anasori B, Gogotsi Y, Anasori B, Gogotsi Y (2019). Introduction to 2D transition metal carbides and nitrides (MXenes). 2D Metal Carbides and Nitrides (MXenes).

[24] Naguib M, Barsoum MW, Gogotsi Yu (2021). Ten years of progress in the synthesis and development of MXenes. Adv. Mater..

[25] Ma C, Yuan Q, Ma M-G, Khalid M, Grace AN, Arulraj A, Numan A (2022). MXenes for electromagnetic interference (EMI) shielding. Fundamental aspects and perspectives of MXenes.

[26] Qing Y, Zhou W, Luo F, Zhou D (2016). Titanium carbide (MXene) nanosheets as promising microwave absorbers. Ceram. Int..

[27] Cao M-S (2019). 2D MXenes: Electromagnetic property for microwave absorption and electromagnetic interference shielding. Chem. Eng. J..

[28] Verma R, Thakur P, Chauhan A, Jasrotia R, Thakur A (2023). A review on MXene and its’ composites for electromagnetic interference (EMI) shielding applications. Carbon.

[29] Jasim SA (2022). MXene/metal and polymer nanocomposites: Preparation, properties, and applications. J. Alloys Comp..

[30] Shahzad F (2016). Electromagnetic interference shielding with 2D transition metal carbides (MXenes). Science.

[31] Liu J (2017). Hydrophobic, flexible, and lightweight MXene foams for high-performance electromagnetic-interference shielding. Adv. Mater..

[32] Nguyen V-T, Min BK, Yi Y, Kim SJ, Choi C-G (2020). MXene(Ti_3_C_2_T_X_)/graphene/PDMS composites for multifunctional broadband electromagnetic interference shielding skins. Chem. Eng. J..

[33] Song P (2020). Honeycomb structural rGO-MXene/epoxy nanocomposites for superior electromagnetic interference shielding performance. SM&T.

[34] Jin X (2020). Flame-retardant poly(vinyl alcohol)/MXene multilayered films with outstanding electromagnetic interference shielding and thermal conductive performances. Chem. Eng. J..

[35] Mičušík M (2023). Aging of 2D MXene nanoparticles in air: An XPS and TEM study. Appl. Surf. Sci..

[36] Machata P (2022). Wettability of MXene films. J. Colloid Interface Sci..

[37] Bekyarova E (2005). Electronic properties of single-walled carbon nanotube networks. J. Am. Chem. Soc..

[38] Lau CH (2008). The effect of functionalization on structure and electrical conductivity of multi-walled carbon nanotubes. J. Nanopart. Res..

[39] Logakis A (2010). Indirect methods for the determination of optimal processing conditions in conductive polypropylene/carbon nanotubes composites. Chem. Phys. Let..

[40] He P (2019). Tailoring Ti_3_C_2_T_x_ nanosheet to tune local conductive network as an environmentally friendly material for highly efficient electromagnetic interference shielding. Nanoscale.

[41] Miao B (2023). Scalable synthesis of 2D Ti_2_CT_x_ MXene and molybdenum disulfide composites with excellent microwave absorbing performance. Adv. Compos. Hybrid Mater..

[42] Gao Q (2021). Flexible multilayered MXene/thermoplastic polyurethane films with excellent electromagnetic interference shielding, thermal conductivity, and management performances. Adv. Compos. Hybrid Mater..

[43] Su SP, Xu YH, China PR, Wilkie CA, McNally T, Pötschke P (2011). Thermal degradation of polymer-carbon nanotube composites. Polymer-Carbon Nanotube Composites: Preparation, Properties and Applications.

[44] Yao L (2021). Partially oxidized Ti_3_C_2_T_x_ MXene-sensitive material-based ammonia gas sensor with high-sensing performances for room temperature application. J. Mater. Sci. Mater. Electron..

[45] Geng H-Z (2008). Effect of carbon nanotube types in fabricating flexible transparent conducting films. J. Korean Phys. Soc..

