# Supplementary material for: Preparation and properties of novel binary and ternary highly amorphous poly(vinyl alcohol)-based composites with hybrid nanofillers
Source: Sci Rep. 2023 Nov 5;13:19126. doi: 10.1038/s41598-023-46083-2 (PMC10625980; doi:10.1038/s41598-023-46083-2)
Supplement: Supplementary file 1 — Supplementary Information. [file 41598_2023_46083_MOESM1_ESM.docx]

**Supplementary Information**

**Preparation and Properties of Novel Binary and Ternary
Highly Amorphous Poly(vinyl alcohol)-based Composites
with Hybrid Nanofillers**

**Anastasiia Stepura^a*^, Matej Mičušik^a^, Federico Olivieri^b^, Gennaro Gentile^b^, Marino Lavorgna^b^, Maurizio Avella^b^, Edita Matysová^c^, Jarmila Vilčáková^d^, Mária Omastová^a*^**

*^a^ Polymer Institute of Slovak Academy of Sciences, Dúbravská cesta 9, 845 41 Bratislava, Slovakia*

*^b^ Institute of Polymers, Composites and Biomaterials of National Research Council of Italy, Via Campi Flegrei, 34, 800 78 Pozzuoli (Naples), Italy*

*^c^ SYNPO akciová společnost, S. K. Neumanna 1316, 530 02 Pardubice V, Czech Republic*

*^d^ Tomas Bata University in Zlín, Faculty of Technology, Vavrečkova 5669, 760 01 Zlín, Czech Republic*

**Corresponding authors. E-mail address: anastasiia.stepura@savba.sk, maria.omastova@savba.sk*

**Figure S1**. XPS of delaminated MXenes of a) C1s region and b) Ti2p region.

**Table S1**. Apparent surface chemical composition of delaminated MXenes as determined by XPS.

| Sample | Surface chemical composition [at. %] | | | | |
| --- | --- | --- | --- | --- | --- |
|  | C1s  Ti_3_C_2_/I/sp^2^/sp^3^/  CO/OCO | O1s  ox/I/II/III | Ti2p  Ti_3_C_2_/I/II/  TiO_2_ | F1s  F^-^/XF/XFX/C-F | Cl2p/N1s/Al2p |
| 2DMX | 30.6  14.6/2.6/4.2/6.0/  1.9/1.2 | 22.1  7.9/2.1/10.4/1.7 | 28.5  11.3/7.6/6.7/2.9 | 12.5  0.3/9.6/1.9/0.7 | 1.9/1.4/3.1 |

*C1s: I – carbide II (or carbide asymmetry)*

*O1s: I – C=O, II – C-O, III - OCO*

*Ti2p: I – Ti^2+^(or carbide asymmetry), II – Ti^3+^*

**Figure S2.** XPS of SWCNTs of a) C1s region and b) Fe2p region.

**Table S2.** Apparent surface chemical composition of SWCNTs as determined by XPS.

| Sample | Surface chemical composition [at. %] | | |
| --- | --- | --- | --- |
|  | C1s  sp^2^/sp^3^/CO/C=O/OCO/Pi-Pi* | O1s  C=O_ar_/C=O_al_/C-O | Fe2p |
| SWCNTs | 97.0  80.9/7.7/0.6/0.6/0.2/7.0 | 1.7  0.7/0.4/0.6 | 1.4 |

| 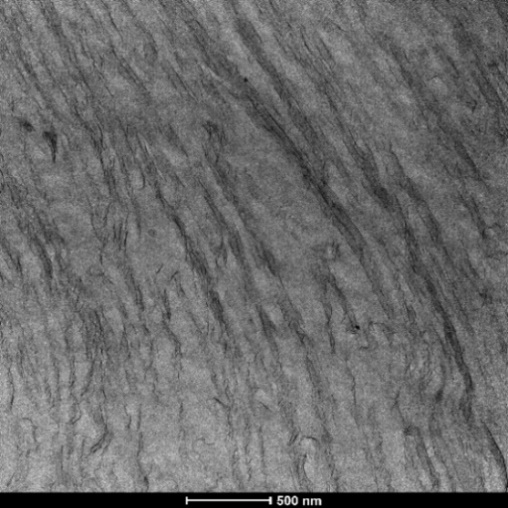  a) | 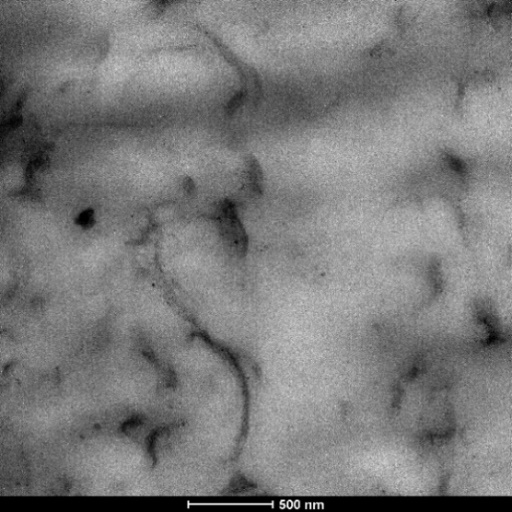  b) |
| --- | --- |
| 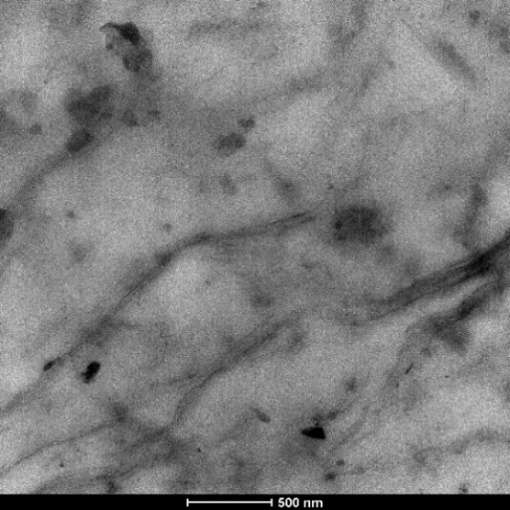  c) | |

**Figure S3.** TEM images of (a) H/3 MX; (b) H/3 SWCNTs; and (c) H/3 SWCNTs/3 MX.

|  |  |
| --- | --- |

а) b)

**Figure S4.** RTA (Reflection, Transmission, Absorption) analysis of all samples at (a) 10 GHz and (b) 11 GHz *(designation of the samples explained in Table 3 in the main text of manuscript).*

**Table S3.** DTA (differential thermal analysis) results of HAVOH composite samples.

| Sample | Residue at 395 °C, % | T_2_, °C  (main peak) | Residue at 895 °C, % |
| --- | --- | --- | --- |
| Neat H (powder) | 39 | 362 | 0.23 |
| Neat H (solv. cast.) | 39 | 347 | 0.22 |
| H/1 MX | 42 | 350 | 0.83 |
| H/3 MX | 48 | 358 | 3.42 |
| H/1 SWCNTs | 32 | 314 | 0.87 |
| H/3 SWCNTs | 37 | 336 | 0.53 |
| H/3 SWCNTs/1 MX | 37 | 315 | 1.97 |
| H/3 SWCNTs/3 MX | 41 | 315 | 4.41 |

**Figure S5.** DTA of thermogravimetric analysis of HAVOH-based polymeric nanocomposites.

**Figure S6.** Plot of TGA (a) T_4_°C degradation step with applied magnification, and (b) magnified T_1_°C degradation region.
